# Supplementary material for: Structural mechanism of DDX39B regulation by human TREX-2 and a related complex in mRNP remodeling
Source: Nat Commun. 2025 Jul 1;16:5471. doi: 10.1038/s41467-025-60547-1 (PMC12216326; doi:10.1038/s41467-025-60547-1)
Supplement: Supplementary file 1 — Supplementary Information [file 41467_2025_60547_MOESM1_ESM.pdf]

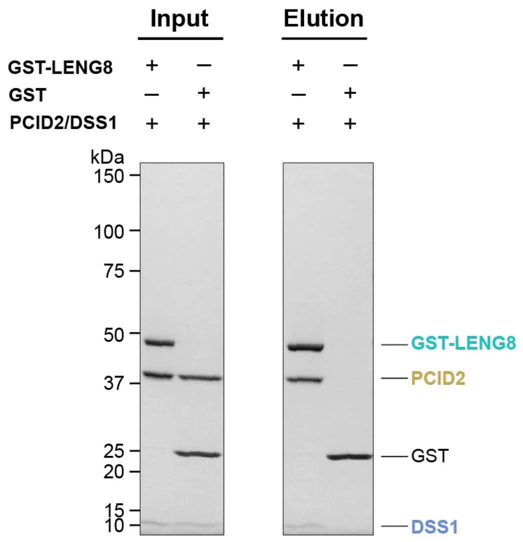


**Supplementary Fig. 1: LENG8 interaction with PCID2/DSS1.**

LENG8 interacts with PCID2/DSS1. In vitro GST pull-down was performed as in Fig. 1b and more samples were loaded on the SDS-PAGE gel for better visualization of DSS1. This experiment has been repeated three times independently with similar results. Source Data are provided as a Source Data file.

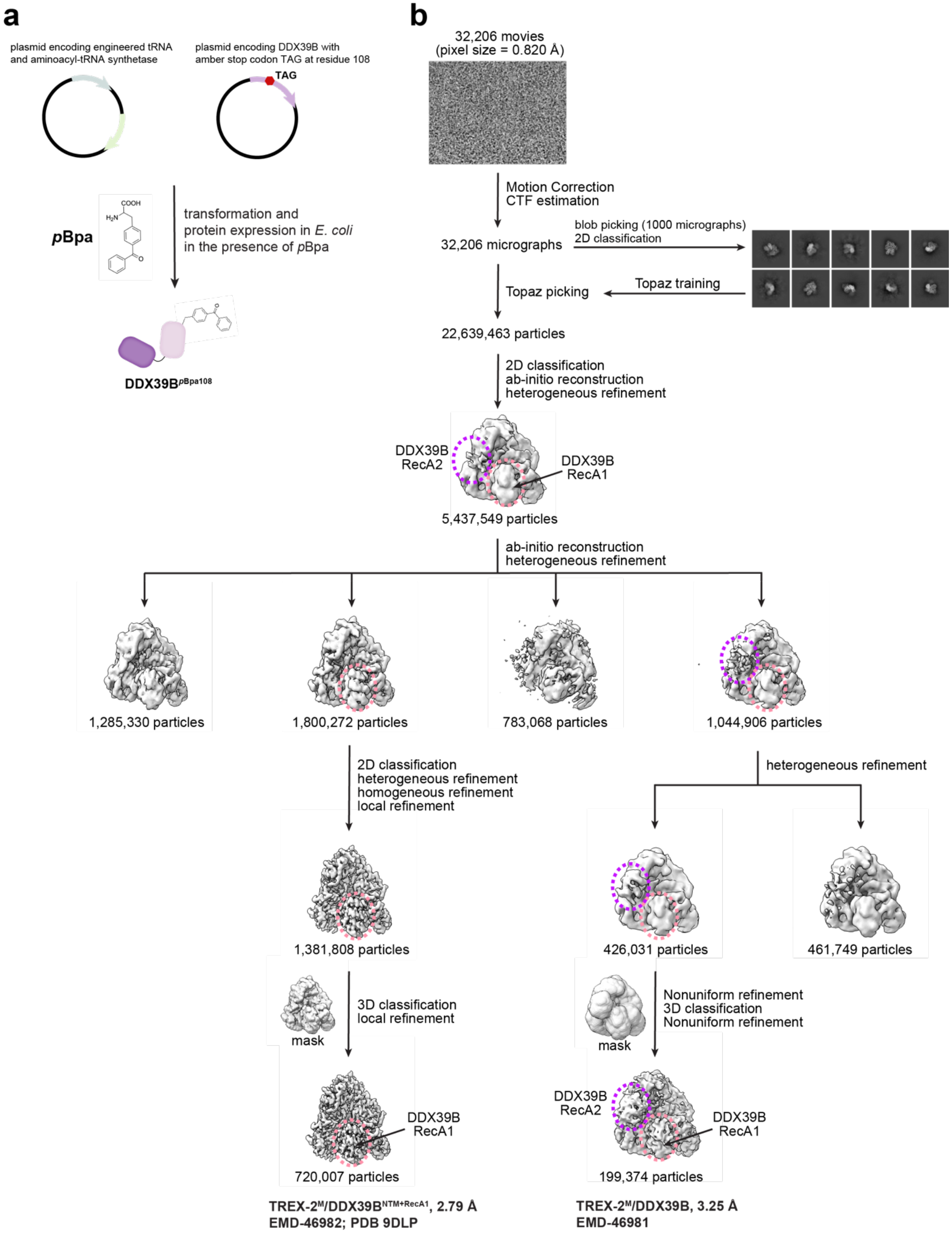
a
plasmid encoding engineered tRNA and aminoacyl-tRNA synthetase
plasmid encoding DDX39B with amber stop codon TAG at residue 108
TAG
pBpa
COOH
H2N
transformation and protein expression in E. coli in the presence of pBpa
DDX39BpBpa108
b
32,206 movies (pixel size = 0.820 Å)
Motion Correction
CTF estimation
32,206 micrographs
blob picking (1000 micrographs)
2D classification
Topaz picking
Topaz training
22,639,463 particles
2D classification
ab-initio reconstruction
heterogeneous refinement
DDX39B RecA2
DDX39B RecA1
5,437,549 particles
ab-initio reconstruction
heterogeneous refinement
1,285,330 particles
1,800,272 particles
783,068 particles
1,044,906 particles
2D classification
heterogeneous refinement
homogeneous refinement
local refinement
heterogeneous refinement
1,381,808 particles
426,031 particles
461,749 particles
mask
3D classification
local refinement
mask
Nonuniform refinement
3D classification
Nonuniform refinement
DDX39B RecA1
720,007 particles
DDX39B RecA2
DDX39B RecA1
199,374 particles
TREX-2M/DDX39BNTM+RecA1, 2.79 Å
EMD-46982; PDB 9DLP
TREX-2M/DDX39B, 3.25 Å
EMD-46981

**Supplementary Fig. 2: Cryo-EM data processing of the TREX-2$^{M}$/DDX39B complex.** (**a**) Schematic for the incorporation of non-natural amino acid *p*Bpa in DDX39B using amber codon suppression[1]. (**b**) Photo-crosslinked TREX-2$^{M}$/DDX39B complex was subjected to cryo-EM studies in the presence of ADP. A total of 32,206 movies were collected on a 300 keV Titan Krios cryo-electron microscope. Data were processed in CryoSPARC. The data yielded a reconstruction of TREX-2$^{M}$/DDX39B map at 3.25 Å resolution (EMD-46981) that contains all domains of DDX39B and a reconstruction of TREX-2$^{M}$/DDX39B$^{NTM+RecA1}$ at 2.79 Å resolution (EMD-46982) that contains the NTM and the RecA1 domain of DDX39B.

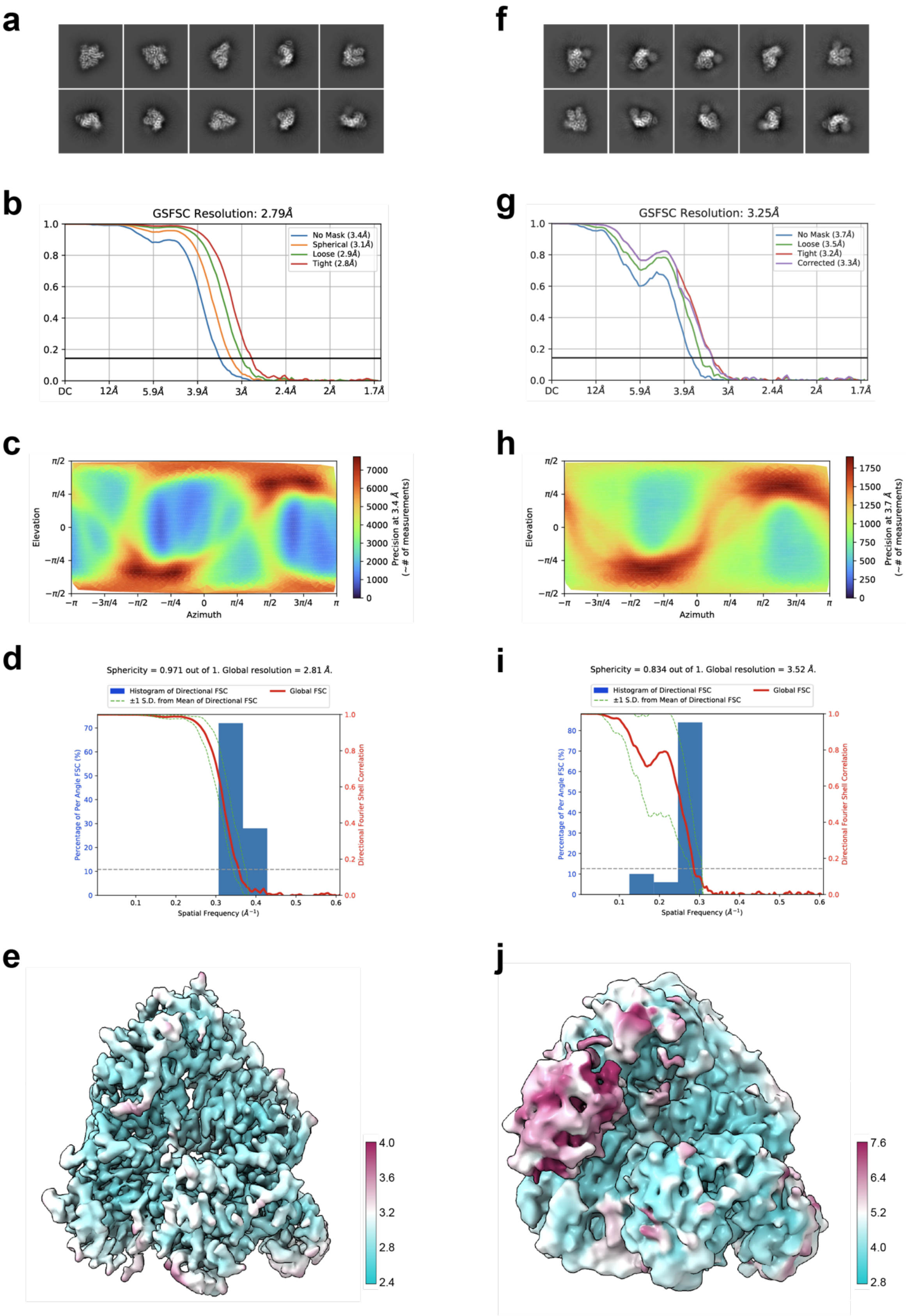

a
f
b
GSFSC Resolution: 2.79Å
No Mask (3.4Å)
Spherical (3.1Å)
Loose (2.9Å)
Tight (2.8Å)
DC
12Å
5.9Å
3.9Å
3Å
2.4Å
2Å
1.7Å
g
GSFSC Resolution: 3.25Å
No Mask (3.7Å)
Loose (3.5Å)
Tight (3.2Å)
Corrected (3.3Å)
c
Elevation
Azimuth
Precision at 3.4 Å (~# of measurements)
h
Precision at 3.7 Å (~# of measurements)
d
Sphericity = 0.971 out of 1. Global resolution = 2.81 Å.
Histogram of Directional FSC
Global FSC
±1 S.D. from Mean of Directional FSC
Percentage of Per Angle FSC (%)
Directional Fourier Shell Correlation
Spatial Frequency (Å$^{-1}$)
i
Sphericity = 0.834 out of 1. Global resolution = 3.52 Å.
e
4.0
3.6
3.2
2.8
2.4
j
7.6
6.4
5.2
4.0
2.8

**Supplementary Fig. 3: Cryo-EM reconstruction of the TREX-2$^{M}$/DDX39B complex.**

(**a-e**) Selective 2D class averages from the final particle set (a), FSC curves (b), particle angular distribution plot (c), 3DFSC analysis (d), and local resolution (e) for TREX-2$^{M}$/DDX39B$^{NTM+RecA1}$ (EMD-46982). (**f-j**) Selective 2D class averages from the final particle set (f), FSC curves (g), particle angular distribution plot (h), 3DFSC analysis (i), and local resolution (j) for TREX-2$^{M}$/DDX39B (EMD-46981).

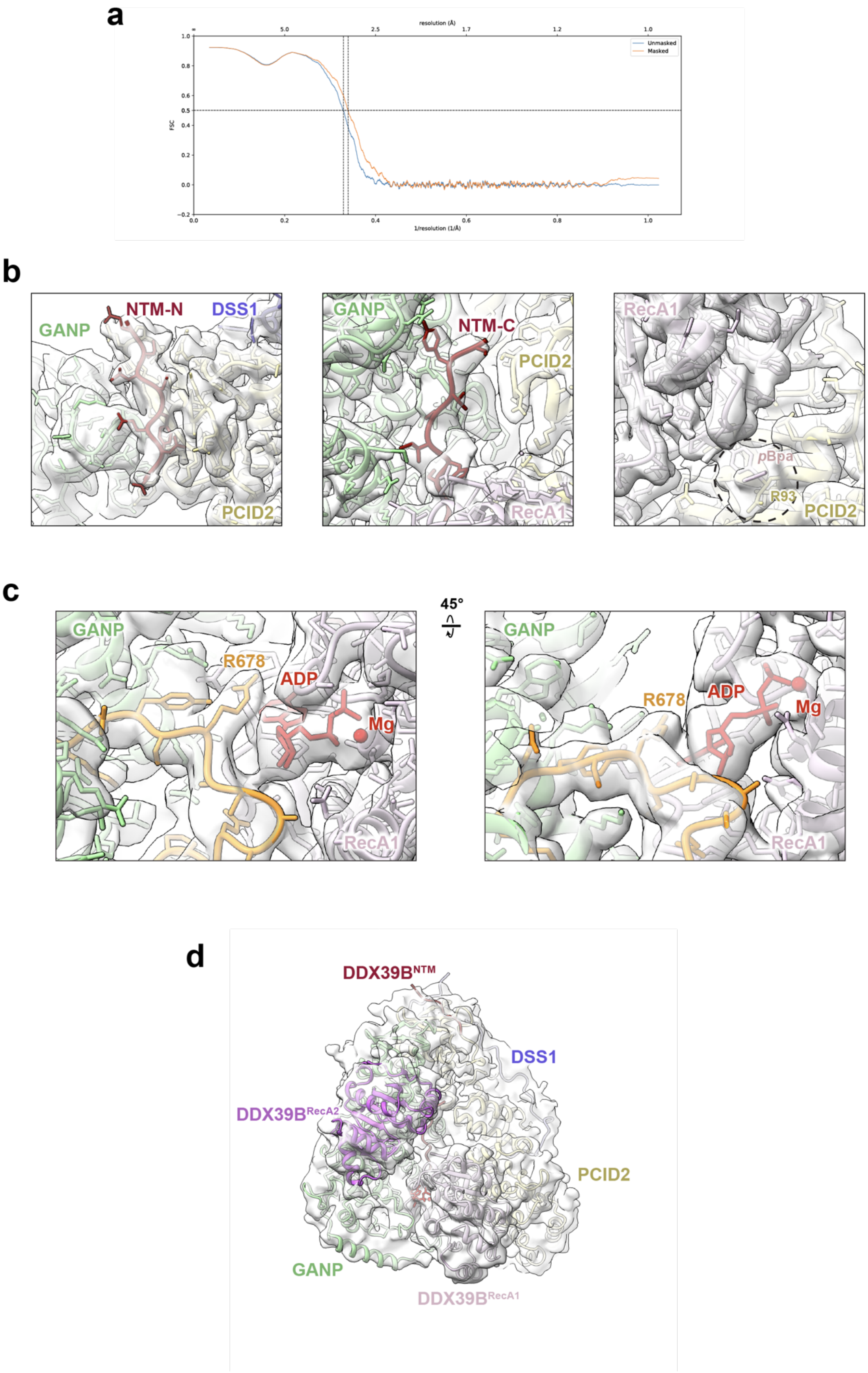

a
resolution (Å)
5.0
2.5
1.7
1.2
1.0
Unmasked
Masked
FSC
1/resolution (1/Å)
b
GANP
NTM-N
DSS1
PCID2
GANP
NTM-C
PCID2
RecA1
RecA1
pBpa
R93
PCID2
c
45°
GANP
R678
ADP
Mg
RecA1
GANP
R678
ADP
Mg
RecA1
d
DDX39BNTM
DSS1
DDX39BRecA2
PCID2
GANP
DDX39BRecA1

**Supplementary Fig. 4: Structural model of the TREX-2$^{M}$/DDX39B complex.**

(**a**) Map versus model FSC curves with or without mask for the TREX-2$^{M}$/DDX39B$^{NTM+RecA1}$ complex (EMD-46982; PDB 9DLP). (**b**) Cryo-EM density at the interfaces with NTM-N (left), NTM-C (middle), and RecA1 domain (right). *p*Bpa incorporated on RecA1 of DDX39B is crosslinked to the R93 residue of PCID2 as indicated at the RecA1 interface. (**c**) Cryo-EM density at the interface of the trigger loop of GANP and the nucleotide binding site of the RecA1 domain of DDX39B in two different orientations. (**d**) Overlay of the TREX-2$^{M}$/DDX39B map (EMD-46981) with the TREX-2$^{M}$/DDX39B model.

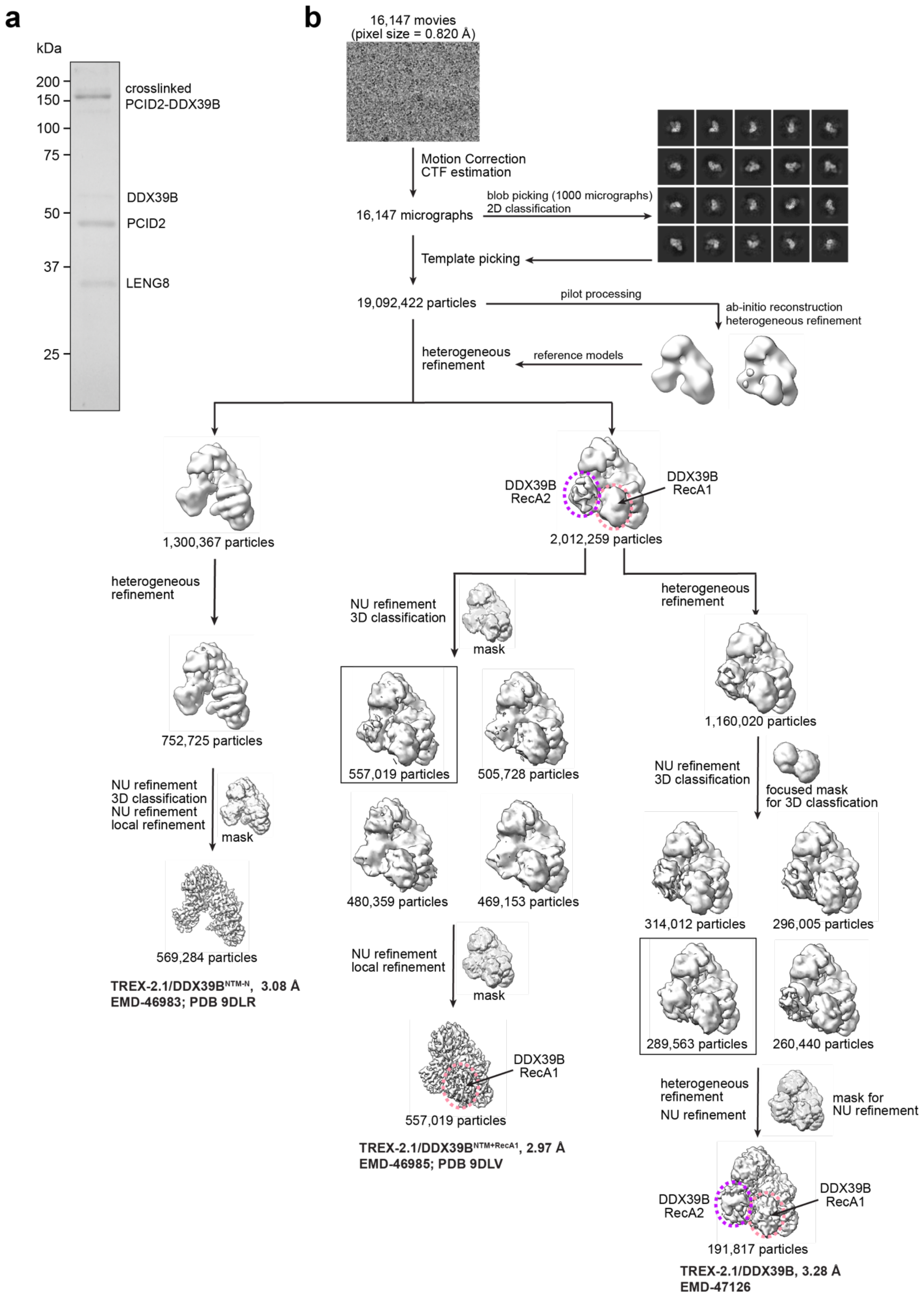
a
kDa
200
150
100
75
50
37
25
crosslinked
PCID2-DDX39B
DDX39B
PCID2
LENG8
b
16,147 movies
(pixel size = 0.820 Å)
Motion Correction
CTF estimation
16,147 micrographs
blob picking (1000 micrographs)
2D classification
Template picking
19,092,422 particles
pilot processing
ab-initio reconstruction
heterogeneous refinement
heterogeneous refinement
reference models
1,300,367 particles
DDX39B RecA2
DDX39B RecA1
2,012,259 particles
heterogeneous refinement
752,725 particles
NU refinement
3D classification
NU refinement
local refinement
mask
569,284 particles
TREX-2.1/DDX39B^NTM-N, 3.08 Å
EMD-46983; PDB 9DLR
NU refinement
3D classification
mask
557,019 particles
505,728 particles
480,359 particles
469,153 particles
NU refinement
local refinement
mask
DDX39B RecA1
557,019 particles
TREX-2.1/DDX39B^NTM+RecA1, 2.97 Å
EMD-46985; PDB 9DLV
heterogeneous refinement
1,160,020 particles
NU refinement
3D classification
focused mask for 3D classfication
314,012 particles
296,005 particles
289,563 particles
260,440 particles
heterogeneous refinement
NU refinement
mask for NU refinement
DDX39B RecA2
DDX39B RecA1
191,817 particles
TREX-2.1/DDX39B, 3.28 Å
EMD-47126

**Supplementary Fig. 5: Cryo-EM data processing of the TREX-2.1/DDX39B complex.** (**a**) Coomassie-stained SDS-PAGE of the photo-crosslinked TREX-2.1/DDX39B complex for cryo-EM studies. This experiment has been repeated three times independently with similar results. (**b**) A total of 16,147 movies were collected on a 300 keV Titan Krios cryo-electron microscope. Data were processed in CryoSPARC. The data yielded reconstructions of three maps including TREX-2.1/DDX39B at 3.28 Å resolution (EMD-47126), TREX-2.1/DDX39B$^{NTM+RecA1}$ at 2.97 Å resolution (EMD-46985), and TREX-2.1/DDX39B$^{NTM-N}$ at 3.08 Å resolution (EMD-46983). Source Data are provided as a Source Data file.

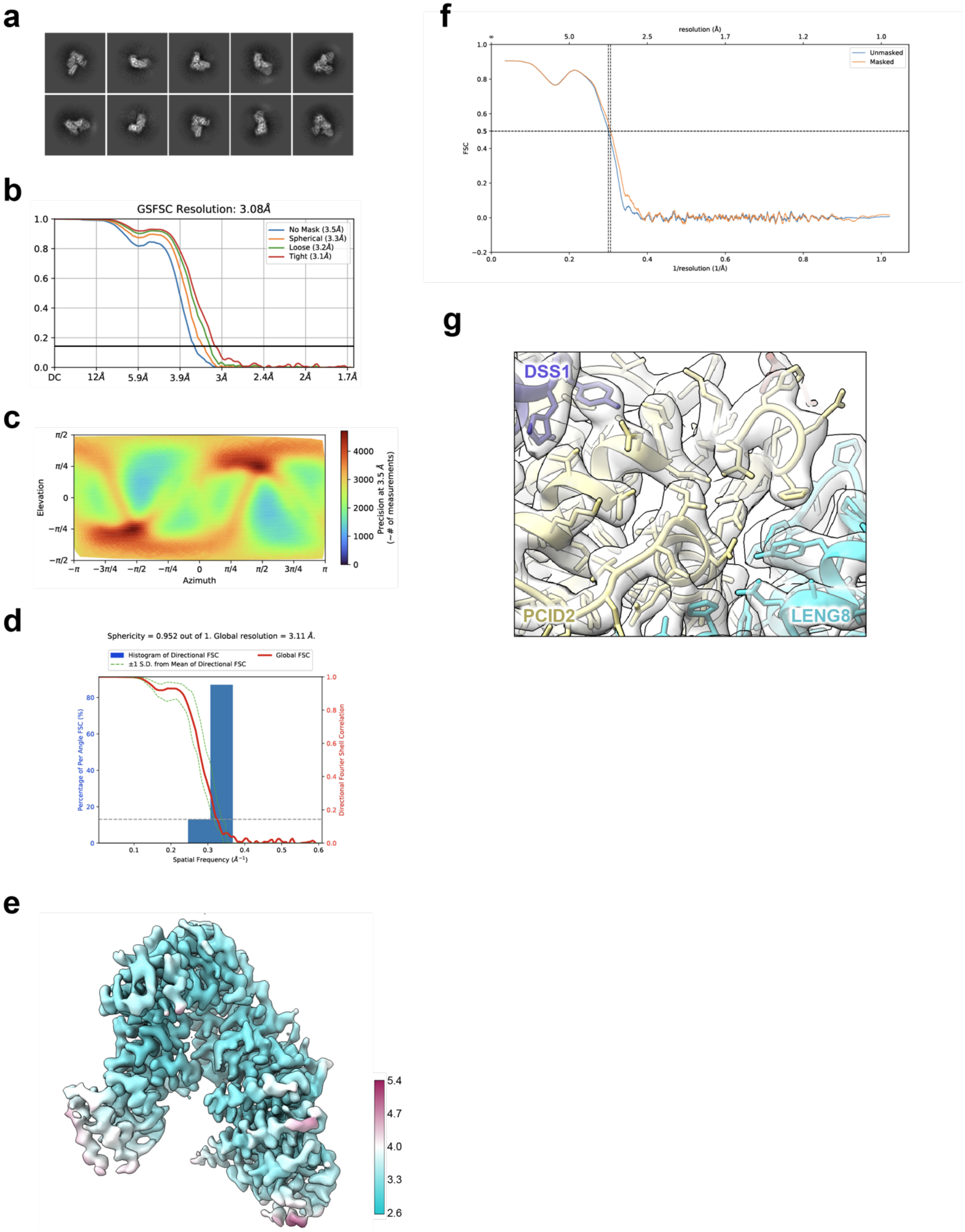
a
b
GSFSC Resolution: 3.08Å
No Mask (3.5Å)
Spherical (3.3Å)
Loose (3.2Å)
Tight (3.1Å)
DC 12Å 5.9Å 3.9Å 3Å 2.4Å 2Å 1.7Å
c
Elevation
Azimuth
Precision at 3.5 Å (~# of measurements)
d
Sphericity = 0.952 out of 1. Global resolution = 3.11 Å.
Histogram of Directional FSC
±1 S.D. from Mean of Directional FSC
Global FSC
Percentage of Per Angle FSC (%)
Directional Fourier Shell Correlation
Spatial Frequency (Å⁻¹)
e
5.4
4.7
4.0
3.3
2.6
f
resolution (Å)
Unmasked
Masked
FSC
1/resolution (1/Å)
g
DSS1
PCID2
LENG8

**Supplementary Fig. 6: Cryo-EM reconstruction and structural model of the TREX-2.1/DDX39B$^{NTM-N}$ complex.**

(**a-e**) Selective 2D class averages from the final particle set (a), FSC curves (b), particle angular distribution plot (c), 3DFSC analysis (d), and local resolution (e) for TREX-2.1/DDX39B$^{NTM-N}$ (EMD-46983). (**f**) Map versus model FSC curves with or without mask (EMD-46983; PDB 9DLR). (**g**) Cryo-EM density at the LENG8-PCID2 interface.

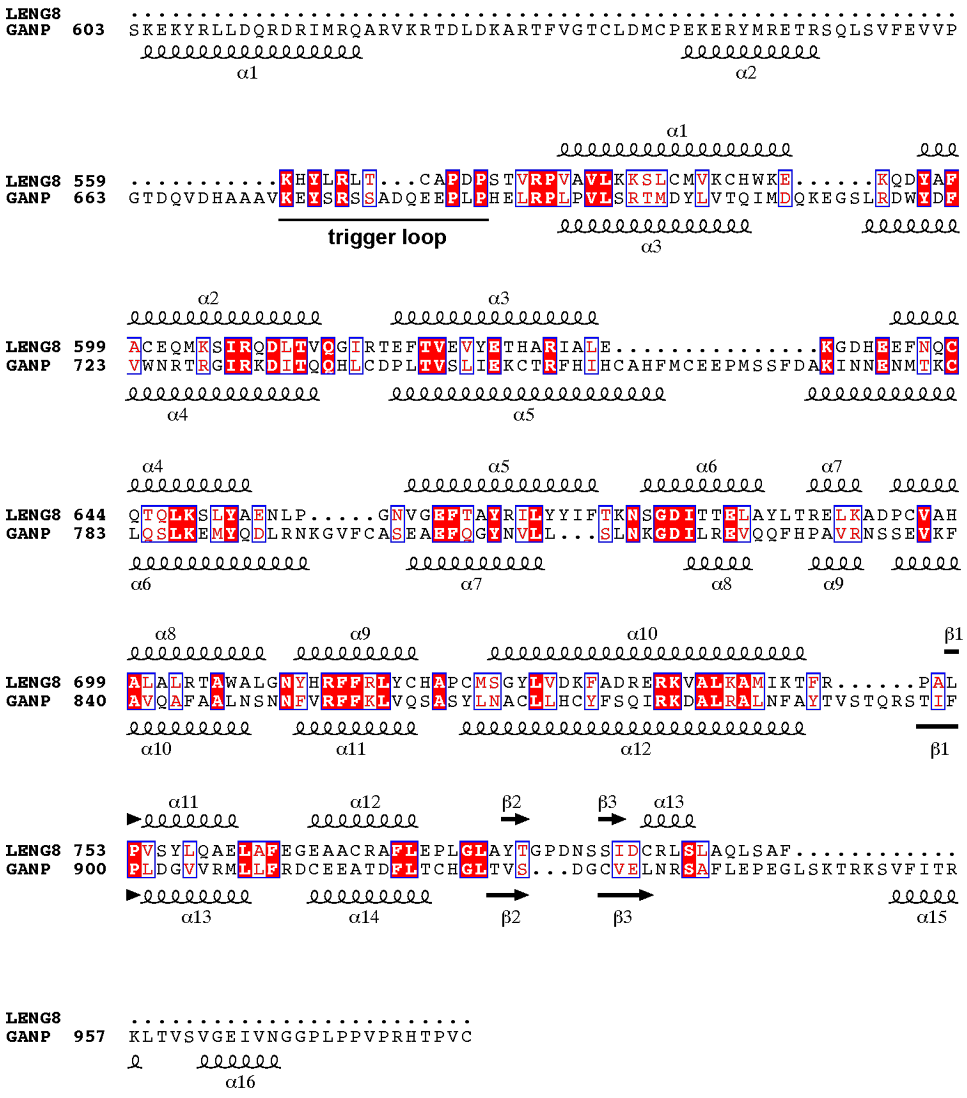


**Supplementary Fig. 7: Structure-based sequence alignment of LENG8 and GANP.** Sequence alignment was guided by the TREX-2$^{M}$/DDX39B$^{NTM+RecA1}$ (PDB 9DLP) and TREX-2.1/DDX39B$^{NTM-N}$ (PDB 9DLR) structures. Secondary structures are indicated at the top (for LENG8) and bottom (for GANP) of the aligned sequences[2].

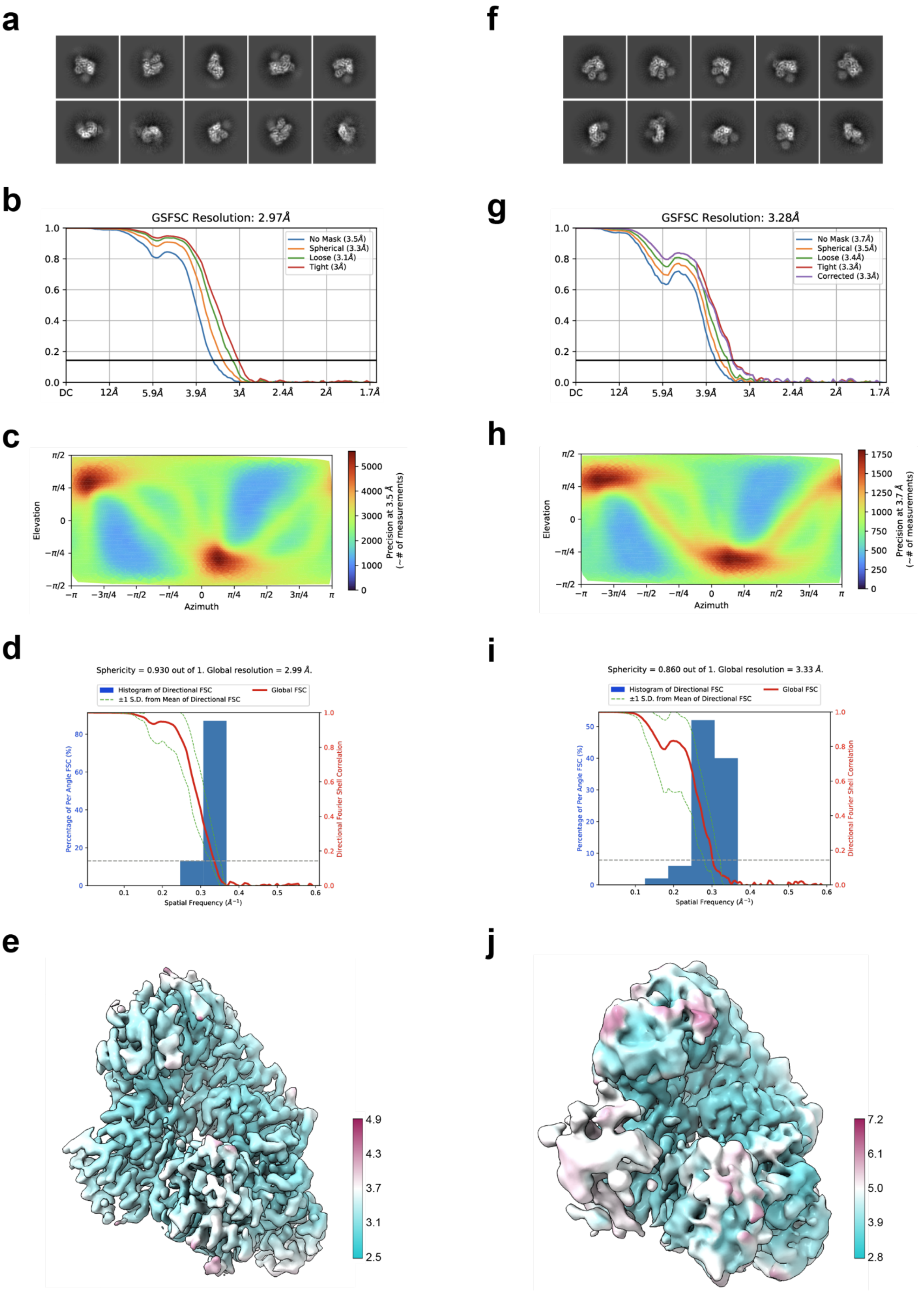
a
f
b
GSFSC Resolution: 2.97Å
No Mask (3.5Å)
Spherical (3.3Å)
Loose (3.1Å)
Tight (3Å)
DC 12Å 5.9Å 3.9Å 3Å 2.4Å 2Å 1.7Å
g
GSFSC Resolution: 3.28Å
No Mask (3.7Å)
Spherical (3.5Å)
Loose (3.4Å)
Tight (3.3Å)
Corrected (3.3Å)
DC 12Å 5.9Å 3.9Å 3Å 2.4Å 2Å 1.7Å
c
Elevation
Azimuth
Precision at 3.5 Å (~# of measurements)
h
Elevation
Azimuth
Precision at 3.7 Å (~# of measurements)
d
Sphericity = 0.930 out of 1. Global resolution = 2.99 Å.
Histogram of Directional FSC
±1 S.D. from Mean of Directional FSC
Global FSC
Percentage of Per Angle FSC (%)
Directional Fourier Shell Correlation
Spatial Frequency (Å⁻¹)
i
Sphericity = 0.860 out of 1. Global resolution = 3.33 Å.
Histogram of Directional FSC
±1 S.D. from Mean of Directional FSC
Global FSC
Percentage of Per Angle FSC (%)
Directional Fourier Shell Correlation
Spatial Frequency (Å⁻¹)
e
4.9
4.3
3.7
3.1
2.5
j
7.2
6.1
5.0
3.9
2.8

**Supplementary Fig. 8: Cryo-EM reconstruction of the TREX-2.1/DDX39B complex.**
(**a-e**) Selective 2D class averages from the final particle set (a), FSC curves (b), particle angular distribution plot (c), 3DFSC analysis (d), and local resolution (e) for TREX-2.1/DDX39B$^{NTM+RecA1}$ (EMD-46985). (**f-j**) Selective 2D class averages from the final particle set (f), FSC curves (g), particle angular distribution plot (h), 3DFSC analysis (i), and local resolution (j) for TREX-2.1/DDX39B (EMD-47126).

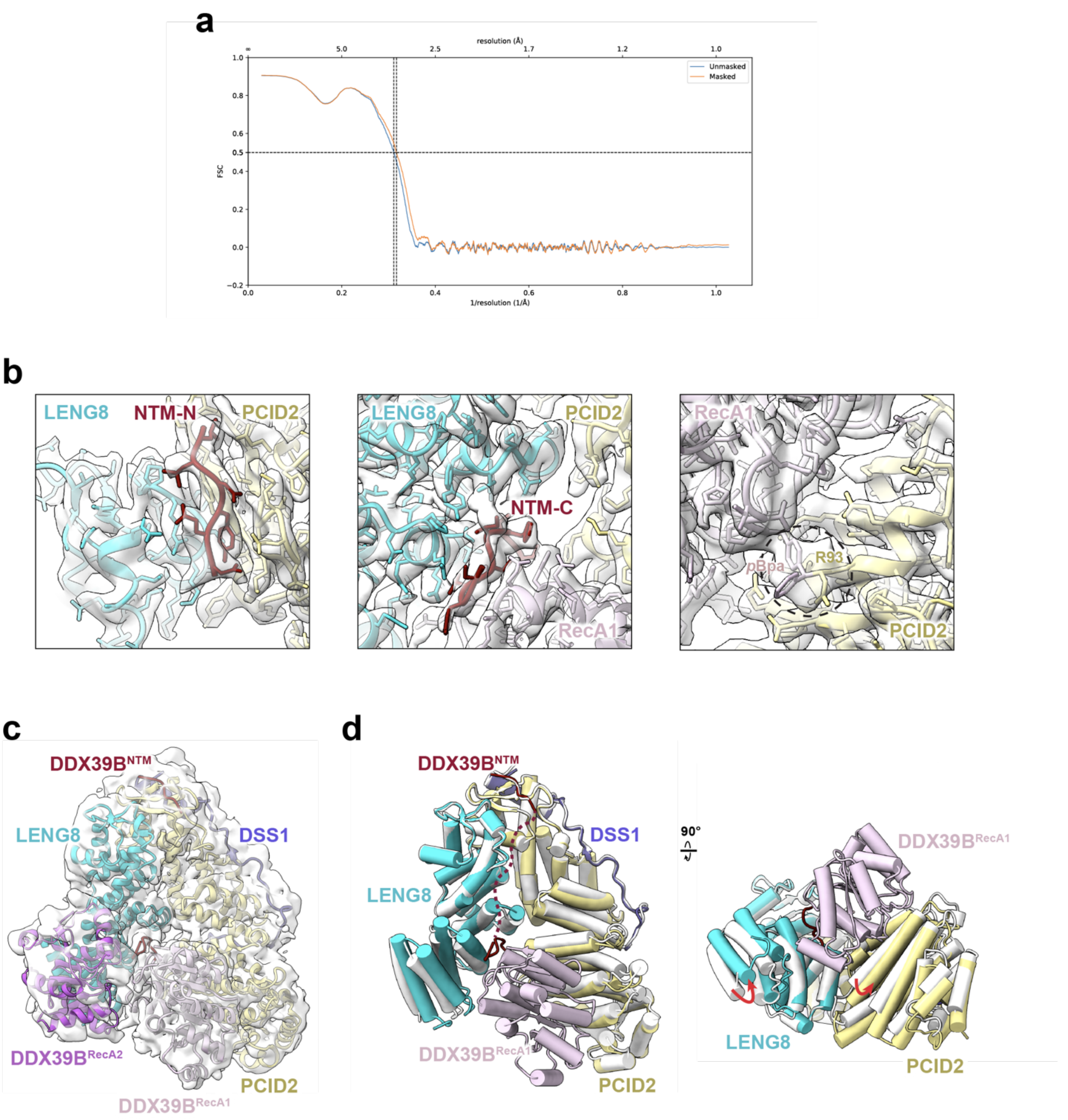


**Supplementary Fig. 9: Structural model of the TREX-2.1/DDX39B$^{NTM+RecA1}$ complex.** (**a**) Map versus model FSC curves with or without mask for the TREX-2.1/DDX39B$^{NTM+RecA1}$ complex (EMD-46985; PDB 9DLV). (**b**) Cryo-EM density at the interfaces with the NTM-N (left), NTM-C (middle), and RecA1 domain (right). *p*Bpa is indicated at the RecA1 interface. (**c**) Overlay of the TREX-2.1/DDX39B map (EMD-47126) with the TREX-2.1/DDX39B model. (**d**) Structural comparison of TREX-2.1/DDX39B$^{NTM+RecA1}$ (PDB 9DLV, colored as in Fig.6d) and TREX-2.1/DDX39B$^{NTM-N}$ (PDB 9DLR, white).

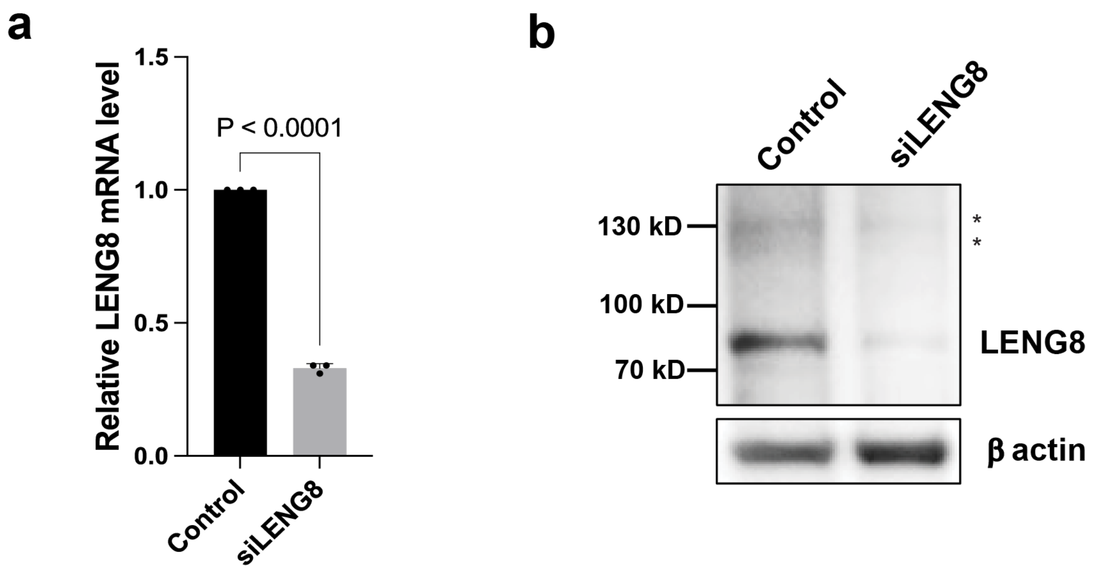


**Supplementary Fig. 10: LENG8 knockdown in A549 cells.**

Cells were transfected with siRNAs targeting LENG8 or control siRNAs at 100 nM for 72 h. (**a**) RNA from whole cell lysates was subjected to qPCR to measure the levels of LENG8 mRNA. The 18S rRNA was used as an internal control. Graphs are mean +/- SD of three independent experiments. *p* values were calculated using unpaired two tailed Student's t-test. (**b**) Whole cell lysates were collected and subjected to western blot analysis to assess the downregulation of LENG8 protein level. Asterisks indicate LENG8 isoforms. β actin was used as loading control. This experiment has been repeated three times independently with similar results. Source Data are provided as a Source Data file.

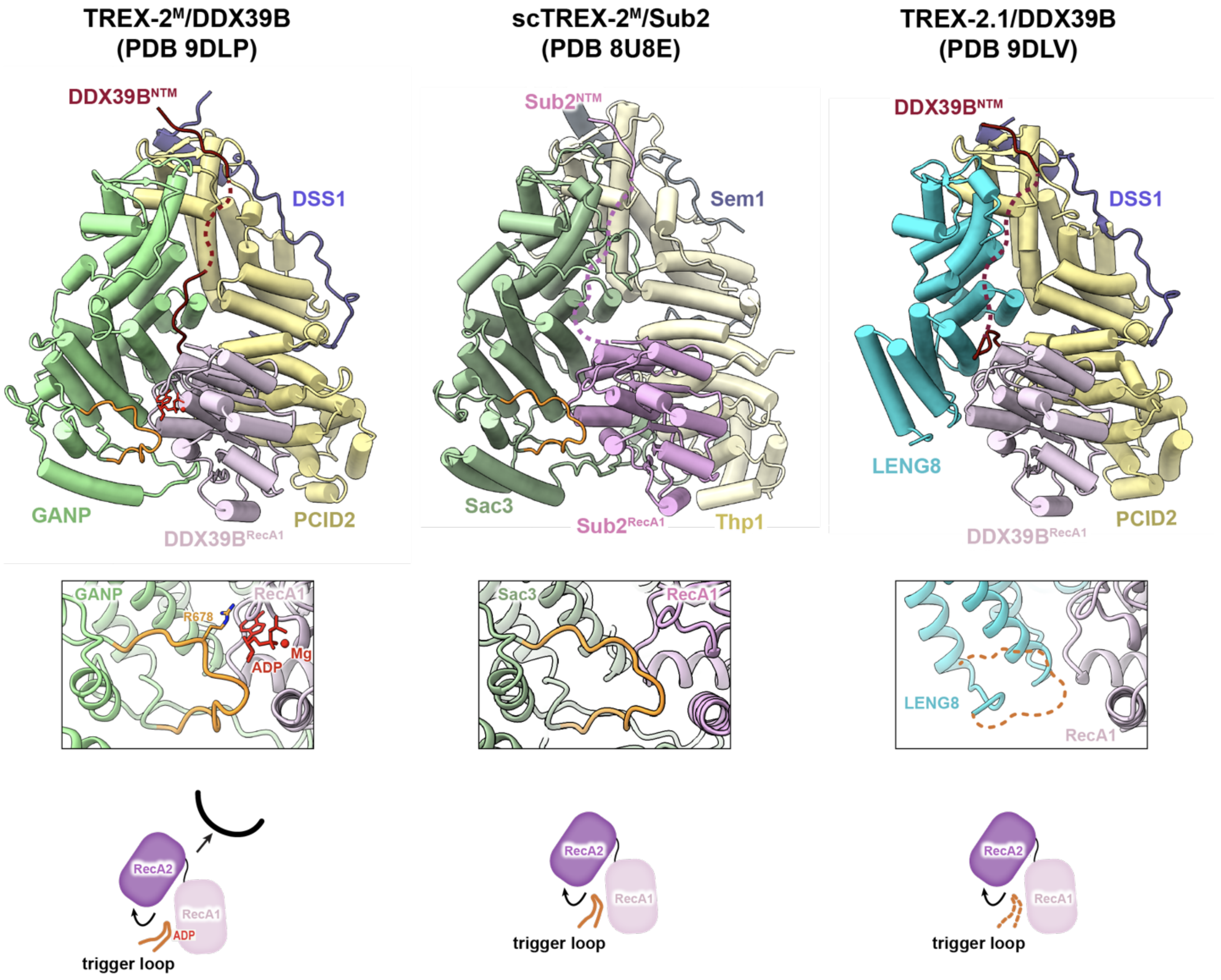


**Supplementary Fig. 11: Structural comparison of TREX-2$^{M}$/DDX39B, TREX-2.1/DDX39B, and the yeast TREX-2$^{M}$/Sub2 complex.** These structures capture different states of the DDX39B/Sub2 ATPase. The trigger loop is colored in orange.

## Supplementary References


1. Chin, J.W., Martin, A.B., King, D.S., Wang, L. & Schultz, P.G. Addition of a photocrosslinking amino acid to the genetic code of Escherichiacoli. *Proc Natl Acad Sci U S A* **99**, 11020-4 (2002).
2. Robert, X. & Gouet, P. Deciphering key features in protein structures with the new ENDscript server. *Nucleic Acids Res* **42**, W320-4 (2014).
