# Supplementary material for: Structural mechanism of DDX39B regulation by human TREX-2 and a related complex in mRNP remodeling
Source: Nat Commun. 2025 Jul 1;16:5471. doi: 10.1038/s41467-025-60547-1 (PMC12216326; doi:10.1038/s41467-025-60547-1)
Supplement: Supplementary file 2 — Description of Additional Supplementary Files [file 41467_2025_60547_MOESM2_ESM.pdf]

Description of Additional Supplementary Files

File Name: Supplementary Data 1

Description: RNASeq data and analysis from results presented in Figure 8. Tab 1: Log_2 value of normalized TPM counts from raw RNAseq data of all mRNAs mapped to the genome in total, nuclear, and cytoplasmic RNA samples of two independent experiments (cells transfected with siRNAs targeting LENG8 or control siRNAs). Tab 2: mRNAs whose total levels are upregulated above 2-fold upon LENG8 knockdown compared to control samples in two independent experiments. Tab 3: mRNAs whose total levels are downregulated less than 0.5-fold upon LENG8 knockdown compared to control samples in two independent experiments. Tab 4: Relative nuclear/cytoplasmic (N/C) ratios between LENG8 knockdown versus control samples of all mRNAs in two independent experiments. Tab 5: mRNAs with a relative N/C ratio over 2 and with total level not substantially altered (between 0.5-fold and less than 2-fold change) in LENG8 knockdown conditions compared to control samples in two independent experiments. Tab 6: RNA features of mRNAs blocked in the nucleus upon LENG8 knockdown compared to control samples.
